# Brugada syndrome diagnosed in a young woman occurring postpartum: case report and literature review

**DOI:** 10.3389/fcvm.2025.1643915

**Published:** 2025-10-10

**Authors:** Wenhui Li, Xiaoyu Pan, Jiaoying Cheng

**Affiliations:** Department of Obstetrics and Gynecology, China-Japan Friendship Hospital, Beijing, China

**Keywords:** Brugada syndrome, pregnancy, delivery, perinatal management, case report

## Abstract

Brugada syndrome (BrS), a hereditary cardiac channelopathy linked to malignant arrhythmias and sudden cardiac death, exhibits significant gender disparity with higher prevalence in males. This case report details a rare occurrence of BrS in a 32-year-old primigravida who suffered an acute cardiopulmonary arrest 2 h postpartum. The patient, with no prior cardiac history or family history of sudden death, underwent an uncomplicated delivery but developed sudden pulselessness post-transfer to the ward. Immediate resuscitation and subsequent evaluations revealed a type I Brugada ECG pattern, while structural cardiac abnormalities, electrolyte imbalances, and other etiologies were excluded. Although genetic testing was negative for pathogenic variants, the clinical diagnosis of BrS was made. An implantable cardioverter-defibrillator (ICD) was implanted for secondary prevention. This case underscores the importance of recognizing BrS in atypical populations, advocating for heightened vigilance in postpartum women with unexplained cardiac events. Further research into female-specific risk factors and collaborative care frameworks is essential to optimize maternal and neonatal outcomes.

## Introduction

Brugada syndrome (BrS) is a hereditary cardiac ion channel disorder associated with malignant arrhythmias and sudden cardiac death in adults with structurally normal hearts (1). The prevalence of BrS varies among different populations, exhibiting significant genetic heterogeneity and geographical differences. The incidence is higher in Asian populations, with statistics showing a prevalence of 12/10,000 in Southeast Asia, compared to only 5/10,000 in Western countries (2, 3). Furthermore, BrS demonstrates a notable gender bias, with a higher incidence in middle-aged men around 40 years of age. The prevalence in males is 10 times higher than that in females (2, 4, 5). Reports of BrS in young women are extremely rare. Here, we report a case of a postpartum woman who unexpectedly suffered a cardiac arrest event, was successfully resuscitated, and later diagnosed with Brugada syndrome. Meanwhile, we review recent literature reports on Brugada syndrome in females to clarify the clinical characteristic manifestations of these patients, and highlight key considerations for clinical management during pregnancy.

## Presentation of case

A 32-year-old primigravida woman was admitted to the maternity ward of China-Japan Friendship Hospital at 36 weeks and 2 days of gestation because of premature rupture of membranes. She had no history of chronic diseases such as heart disease or hypertension. This was her first pregnancy and she had been regularly attending prenatal check-ups at our hospital. Non-invasive DNA testing during pregnancy showed low risk. At 24 weeks of gestation, an oral glucose tolerance test was performed, and she was diagnosed with gestational diabetes mellitus. Dietary modifications, exercise, and fingerstick glucose monitoring were prescribed. Her blood glucose levels were well controlled without the need for insulin treatment. The results of all other prenatal screening tests were normal, including electrocardiogram (Supplementary Material S1) and blood pressure. The blood type was O, Rh-positive, with negative antibody testing.

**Figure 1:**
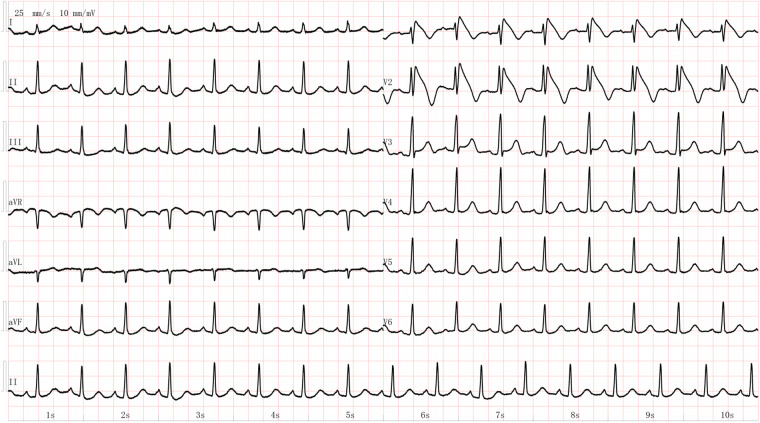
The electrocardiogram of the young female patient showing Brugada waves after achieving recovery of spontaneous circulation.

In hospital, the patient felt occasional contractions and appeared well on all examinations. She was admitted to the labor and delivery unit for observation and oxytocin was administered for labor induction. Epidural labor analgesia was performed after the onset of labor at 4:00 the next day, with a combination of 0.1% ropivacaine and 3 µg/ml fentanyl, and she delivered vaginally at 8:32 with a smooth process. The total duration of labor lasted 7 h and 38 min, with the first, second, and third stages being 7 h, 32 min, and 6 min respectively. Then Oxytocin was routinely used to facilitate uterine contraction and prevent postpartum hemorrhage. The blood loss was approximately 200 ml. The newborn's Apgar scores at 1, 5, and 10 min were all 10 points. After delivery, the patient continued to have electrocardiogram (ECG), blood pressure and oxygen saturation monitoring in the delivery room for nearly 2 h. She experienced mild palpitations but all vital signs remained stable. Then the patient was planned to transfer to the ward. During this period, she was fully conscious and answered questions appropriately but complained of a sensation of “premature beat”. At 10:17, she suddenly experienced a severe coughing episode accompanied by upward deviation of the eyes and marked facial pallor. Concurrently, the patient was noted to be unresponsive and pulseless. Cardiopulmonary resuscitation was begun and the cardiac monitor revealed pulseless ventricular tachycardia. After 5 min of chest compression and a 200J defibrillation shock, a transient return of spontaneous circulation was achieved, with a heart rate of 120–150 bpm. Random blood glucose was normal. The patient was afebrile throughout her hospital stay, including during and after the cardiac event. Endotracheal intubation was performed, and mechanical ventilation was initiated. The patient was transferred to the Intensive Care Unit (ICU) for further treatment. Her hemodynamic parameters gradually stabilized. A comprehensive workup, including electrolytes, thyroid function, myocardial enzyme tests, CT scanning of brain and chest, as well as transthoracic echocardiography, was unremarkable. Serial ECGs showed a typical Brugada type 1 ECG pattern (Figure 1). A multidisciplinary consultation was promptly organized to assist in the diagnosis and formulation of the treatment plan for the patient.

The differential diagnosis was rapidly constructed to identify the underlying cause. The patient had no significant medical or family history. Her prenatal check-ups and delivery process were uneventful. There was no special medication used during delivery. She did not experience cough, dyspnea, significant elevation of blood pressure, abnormal coagulation function, severe bleeding, or other neurological abnormalities. The cardiac arrest occurred 2 h after delivery. Comprehensive evaluations of the cardiovascular, neurological, and respiratory systems revealed no severe organic or functional abnormalities. A transient elevation of cardiac biomarkers followed by prompt normalization is considered to reflect short-lived myocardial injury and ischemia precipitated by the acute cardiac event and electrical defibrillation. Based on the patient's history and examination results, conditions such as amniotic fluid embolism, anaphylactic shock, eclampsia, epilepsy, cerebrovascular diseases and pulmonary embolism were excluded. A diagnosis of Brugada Syndrome was considered and genetic testing was performed to confirm it. The patient later had an implantable cardioverter defibrillator (ICD) implanted, and her condition stabilized soon before discharge and in the follow-ups. The discharge diagnoses were Brugada syndrome, Hypoxic-ischemic encephalopathy, Post-resuscitation after cardiac arrest, Post-ICD implantation, Gestational diabetes mellitus and Post-vaginal delivery. Subsequently genetic tests did not find a clear positive mutation correlated to Brugada Syndrome (Supplementary Material S2).

## Discussion

### Epidemiological characteristics and etiology of Brugada syndrome in women

Cardiovascular diseases are one of the leading causes of maternal mortality. In recent years, the inpatient mortality rate for patients with cardiovascular disease during delivery hospitalization has increased dramatically, with a 14–20 times higher death risk from 1995 to 2006 (6). In 2024, the mortality rate from cardiovascular diseases was approximately 0.5% among nearly 400,000 childbirth hospitalizations in the United States (7). Reports about the pregnancy risks and outcomes of women with Brugada syndrome are extremely limited. However, severe cardiac events during pregnancy and the perinatal period seems uncommon in Brugada syndrome patients.

Brugada syndrome was first defined in 1992 as a clinical syndrome characterized by right bundle branch block, ST-segment elevation, and sudden cardiac death. Patients typically exhibit ventricular arrhythmias and susceptibility to sudden cardiac death without obvious structural heart disease, along with specific ECG findings. Genotype-phenotype studies have shown that abnormalities in voltage-gated ion channels or reduced cell surface molecular expression leading to decreased peak sodium current are common electrophysiological markers of BrS (8). Loss-of-function mutations in the voltage-gated cardiac sodium channel gene SCN5A are the most common genetic cause of BrS, though it is only found in about 20% of cases (1), and seems to be more prevalent among females (9, 10). The gender differences in the phenotype of BrS are more pronounced than in other autosomal dominant inherited arrhythmia syndromes. Some studies suggest that this gender-related difference may be influenced by hormones (11).

Research has shown that testosterone levels in male BrS patients are significantly higher than in normal males, suggesting that hormones may modulate ion currents. In populations receiving prostate cancer treatment, patients diagnosed with BrS had the characteristic type I ECG pattern disappear after castration (12). Additionally, Di Diego et al. conducted studies on isolated right ventricular epicardial cells from male and female dogs, showing significant differences in transient outward potassium current (Ito) density and deactivation kinetics, supporting the idea of a gender-related cellular and ionic basis. They proposed that the more prominent Ito in males forms the basis for their tendency to develop the Brugada phenotype. Estrogen inhibits the expression of the gene Kv4.3, which encodes the Ito current, while testosterone enhances outward currents and reduces inward currents, exerting opposite effects. Animal experiments also confirmed that increased estrogen levels lead to downregulation of Kv4.3 expression in late pregnancy rats, resulting in reduced Ito in the myometrium, which partially explains the lower incidence in women (12–14). However, it is a limitation of this case report that hormone levels were not measured.

### Diagnosis of Brugada syndrome

The diagnosis of Brugada syndrome is mainly based on several factors:

1. ECG: The initial diagnosis of Brugada syndrome is based on the characteristic ECG pattern, specifically the type I Brugada ECG pattern. In this pattern, there is significant ST-segment elevation in leads V1 and V2 (sometimes extending to V3), with a QRS complex resembling right bundle branch block. The ST-segment is dome-shaped and continues to an inverted T-wave. The Type I Brugada pattern is the only pattern considered diagnostic.
2. Family History: A family history of sudden cardiac death, particularly in individuals under 45 years of age or with similar dome-like changes in ECG patterns, plays a critical role in diagnosis.
3. Provocation Tests: When the baseline ECG is non-diagnostic in patients with suspicious symptoms or a positive family history, or when concealed forms are suspected, drug provocation tests are needed to induce characteristic ECG changes. Commonly used drugs include ajmaline, procainamide, flecainide, or pilsicainide.
4. Genetic Testing: Genetic testing for SCN5A gene mutations, which encode the α-subunit of the cardiac sodium channel, is often used to confirm the diagnosis of Brugada syndrome.
5. Clinical Manifestations: These may include syncope, near-death breathing patterns during sleep, documented ventricular fibrillation, or self-terminating polymorphic ventricular tachycardia.
6. Exclusion of Other Factors: Other potential causes of abnormal ECG, such as myocardial ischemia, electrolyte imbalances, or metabolic disorders, need to be ruled out. The diagnosis of Brugada syndrome involves a comprehensive evaluation that includes ECG findings, family history, genetic testing, and clinical symptoms.

In this case, the patient had no significant medical history, denied arrhythmia, cardiovascular diseases, or a specific family history. Early pregnancy ECG was normal. She had gestational diabetes, which was well-controlled with diet and exercise. After premature rupture of membranes, she underwent induced labor, and the delivery was uncomplicated with normal fetal heart monitoring and minimal vaginal bleeding. The patient had no allergies, and after 2 h of observation, she suddenly experienced a cardiac arrest. There were no signs of anaphylactic shock, amniotic fluid embolism, or pulmonary embolism. ECG showed the typical type I Brugada pattern, and further tests showed no significant abnormalities. Although there's no significant genetic mutations, the diagnosis of Brugada syndrome was considered. An implantable cardioverter- defibrillator (ICD) was implanted to prevent repeat cardiac arrest in future. However, it should also be acknowledged that the diagnosis in this case was primarily based on the patient's cardiac arrest episode and transient abnormal electrocardiogram (ECG) findings. Whether there exist other undetected underlying factors that could have contributed to the arrhythmia and subsequent cardiac arrest remains undetermined.

### Risk assessment in Brugada syndrome

Over the past few decades, clinical symptoms, ECG markers, and genetic markers of Brugada syndrome have been extensively studied. Researchers are dedicated to identifying high-risk factors that can assess the occurrence of new or recurrent cardiac events in asymptomatic Brugada-type ECG or BS syndrome patients, with the aim of focusing on managing high-risk patients to prevent the serious consequences. Beyond the rarity of this clinical presentation, the well-documented ECG in this case revealed a remarkable coved-type J-point elevation exceeding 0.30 mV. This is not merely a diagnostic marker but potentially a quantitative indicator of real-time electrical instability. Therefore, the profound ST-segment elevation observed in our patient during a period of extreme physiological stress (postpartum) may represent a transient but critical amplification of this arrhythmogenic substrate, unmasking the full extent of her arrhythmic risk. This observation consistent with previous studies, supports the hypothesis that dynamic changes in J-point amplitude could serve as a powerful, real-time prognostic marker for imminent arrhythmic events (15). Patients with BrS and inferolateral J-point elevation appeared to have a worse overall outcome, a coved-type ST-segment elevation was believed as an independent predictor for subsequent events. Moreover, family history of sudden death, a spontaneous change in ST segment, syncopal episodes, and late potentials also had significant values to detect a high-risk group (16). Beyond ECG markers and clinical symptoms, other tools are sometimes used for risk assessment. Signal-averaged ECG (SAECG) may identify late potentials, while invasive electrophysiological (EP) testing with programmed ventricular stimulation can stratify inducibility. Implantable cardiac monitors (ICMs) allow long-term rhythm surveillance, particularly in equivocal cases.

Several risk scoring models have been developed globally, such as the Prediction of Arrhythmic Events (PAT) score (17), BRUGADA-RISK score (18), Shanghai Score System (19), and Sieira Score (20). However, these existing models have proven inadequate in accurately predicting future ventricular fibrillation (VF) events in BrS patients. Therefore, risk stratification for BrS remains challenging, particularly for patients in the intermediate-risk category. Although the condition primarily affects males, with a higher risk of arrhythmic events, females have received less attention in the research (21, 22). It is well-established that symptomatic patients have a higher risk of sudden cardiac death compared to asymptomatic patients, and this relationship is more pronounced in males. However, studies indicate that this association may not be as prominent in females. Some reports suggest no significant difference in arrhythmia risk between symptomatic and asymptomatic female patients. Moreover, the risk of sudden cardiac death in women is often underestimated due to a lack of research on female-specific risk factors and multi-parameter risk scoring systems. Therefore, global clinical studies on female BrS patients are needed to develop gender-specific risk stratification methods for asymptomatic patients.

### Pregnancy and Brugada syndrome

Reports on pregnancy and Brugada syndrome are extremely limited. We conducted an electronic search in PubMed (March 2025) with the use of the following MesH terms: Brugada syndrome, pregnancy, parturition, labor, and delivery at primary studies that reported on or investigated pregnancy and/or delivery in women affected by Brugada syndrome and the management of these patients. After excluding the duplicate cases, we identified 15 women with BS who experienced pregnancy or childbirth in 15 articles (23–37). Most patients were diagnosed before pregnancy, an only 2 of these patients were firstly diagnosed with BS during the perinatal period (Table 1). To our knowledge, this is the first case documenting such a significant, real-time ECG change concurrently with a cardiac arrest in a postpartum BrS patient. The timing of the event, occurring 2 h postpartum, suggests a potential multifactorial trigger. The cumulative physiological stress of labor, the abrupt postpartum fall in estrogen levels, and the potential pro-arrhythmic properties of ropivacaine used for epidural analgesia may have acted synergistically to unmask an underlying arrhythmogenic substrate in this predisposed patient.

**Table 1:** Characteristics of two patients with BS diagnosed during the perinatal period.

Patient	Age	Proband/family member	Time of diagnosis	*Symptom*	*SCN5A* DNA testing	Brugada syndrome	ICD	Delivery mode	Events during delivery
1 (26)	18	Proband	Postparm	Abrupt loss of consciousnes 1 week postpartum associated with a fever of mastitis	Not mentioned	Spontaneous Brugada Type 1 pattern on ECG	ICD implanted after delivery	Not mentioned	No
2 (27)	24	Proband	Postpartum	Two episodes of seizures in sleep during pregnancy	No	Asymptomatic monomorphic ventricular ectopy originating from the right ventricular outflow tract since childhood	ICD implanted after delivery	Not mentioned	No
				Nocturnal agonal respiration after childbirth		Positive ajmaline provocation test after delivery			

ECG, electrocardiogram; ICD, internal cardioverter defibrillator.

During pregnancy, significant changes occur in maternal cardiac output and blood flow, with cardiac output increasing by 30%–50% and resting heart rate rising by 10–20 bpm. In the process of labor, adrenergic stimulation may trigger cardiac events (30). Patients with Brugada syndrome typically exhibit vagally-mediated arrhythmias or nighttime cardiac arrest, and their resting ECG often shows no significant or only marginal abnormalities. The Brugada-type ECG pattern may appear transiently or spontaneously during drug administration or febrile stimuli. The analgesic and anesthetic drugs used during childbirth, especially sodium channel blockers (such as lidocaine, bupivacaine, etc.), may affect the ECG of patients with Brugada syndrome, potentially leading to the appearance of the Brugada-type ECG pattern. These anesthetic agents exert their effects by inhibiting sodium channels, which may increase the risk of arrhythmias in patients with Brugada syndrome. In particular, local anesthetics can induce ST-segment elevation, especially when administered in high doses or when their effects are prolonged, which may lead to arrhythmias or exacerbate the condition. The cardiotoxicity of ropivacaine lies between that of lidocaine and bupivacaine. When applied during labor analgesia in this patient with Brugada syndrome, it may potentially induce arrhythmias to some extent.

Although a few studies have shown that the risk of arrhythmias in Brugada syndrome patients during pregnancy does not increase, it is still recommended to avoid the use of high doses or long-acting local anesthetics, and to adopt multidisciplinary management. If their use is necessary, it should be done under the guidance of a cardiology specialist, with continuous ECG monitoring. For patients diagnosed with Brugada syndrome, a thorough evaluation should be performed prior to childbirth, and an individualized pain management plan should be developed in collaboration with anesthesiologists, cardiologists, and obstetric specialists to minimize the risk of arrhythmias. Perinatal care and delivery plans should involve the participation of cardiologists and genetic experts to ensure the safety of both mother and child (38, 39).

## Conclusion

Women with Brugada syndrome might imply a low overall risk to develop cardiac arrhythmias during pregnancy. But effective risk stratification and assessment were lacking. Further research on multi-parametric risk scores based on female cohorts is warranted. A thorough evaluation should be performed before pregnancy and prior to childbirth, and an individualized management plan should be developed in collaboration with anesthesiologists, cardiologists, and obstetric specialists to minimize the risk of arrhythmias and ensure the safety of mothers as well as the newborn children.

## Data Availability

The original contributions presented in the study are included in the article/Supplementary Material, further inquiries can be directed to the corresponding author.

## Supplementary material

Supplementary Data Sheet 1 The baseline antenatal ECG.

Supplementary Figure 1 The genetic test.
