# Supplementary material for: Brugada syndrome diagnosed in a young woman occurring postpartum: case report and literature review
Source: Front Cardiovasc Med. 2025 Oct 10;12:1643915. doi: 10.3389/fcvm.2025.1643915 (PMC12549572; doi:10.3389/fcvm.2025.1643915)
Supplement: Supplementary Data Sheet 1 — The baseline antenatal ECG. [file Datasheet1.pdf]

BGI Shenzhen BGI Medical Inspection Laboratory

Sample ID: XX [redacted]

Whole Exome Sequencing Test Report (Singleton)

Hospital Information			
**Referring Hospital**	**Referring Physician**	**Outpatient/Inpatient ID**	**Department**
-	-	-	-

Sample Information							
**Date Received**	**Sample ID**	**Sample Type**	**Name**	**Relationship**	**Sex**	**Ethnicity**	**Date of Birth/Age**
2024-11-09	XX	Whole Blood	XX	Proband	Female	Han	1992-03-12/32
**Clinical Presentation and Family History**	XX	Confirmed patient; Syncope, Cardiorespiratory arrest, Post CPR, Onset at 32 years. Brugada syndrome? Gestational diabetes mellitus. No family history of genetic disorders.					
**Phenotype Matched Standard Terminology**	-/-						
**Clinically Suspected Disease/ Focused Analysis Gene Set**	-/-						

Test Information			
**Test Item**	Clinical Whole Exome Sequencing - Singleton (Category B)	**Test ID**	
**Target Region**	Exonic regions of approximately 20,000 genes in the human genome + Mitochondrial genome		
**Testing Strategy**	Analysis of genes with clear pathogenic relationships recorded in the OMIM database, based on the proband's chief complaint.		
**Testing Method**	Array-based capture high-throughput sequencing		

Test Conclusion

Primary Findings:
One variant of uncertain significance (VUS), partially related to the proband's phenotype, was detected in the *MYL3* gene associated with Familial Hypertrophic Cardiomyopathy type 8.

Secondary Findings:
No other variants related to the clinical presentation were detected.

Mitochondrial Gene Test Results:
No mitochondrial gene variants related to the clinical presentation were detected.

Large CNV Test Results:
No pathogenic or likely pathogenic chromosomal CNV variants related to the proband's phenotype were detected.

Incidental Findings:
No pathogenic or likely pathogenic incidental variants were detected (SecondaryFinding_Var database).

Primary Findings

Gene	Chromosomal Position	Transcript Nucleotide Change (Amino Acid Change)	Gene Region	Genotype	Pathogenicity Classification	Associated Disease/ Inheritance Pattern	References
MYL3	chr3:46901000	NM_000258.2:c.446T>C (p.Met149Thr)	EX4/ CDS4	Heterozygous	Variant of Uncertain Significance (VUS)	Familial Hypertrophic Cardiomyopathy type 8 (OMIM:608751)/AD, AR	[1-4]
**Inheritance Patterns: AD denotes Autosomal Dominant, AR denotes Autosomal Recessive, XL denotes X-Linked, YL denotes Y-Linked, UNK denotes Unknown inheritance pattern.<br>**Primary Findings include: Pathogenic/Likely Pathogenic variants related to the clinical phenotype; Variants of Uncertain Significance (VUS) related to the clinical presentation with a consistent inheritance pattern.							

Result Interpretation:

1. The variant *MYL3*; NM_000258.2:c.446T>C(p.Met149Thr) was detected. Relevant reports exist for this variant [1-4]. According to the ACMG guidelines (Appendix), this variant is classified as a Variant of Uncertain Significance (VUS), PM2_Supporting. Evidence items are as follows:
PM2_Supporting: Downgraded PM2 condition.
Population Frequency (Database versions see Appendix):

Database	1000 Genomes	ESP6500	ExAC	GnomAD	GnomAD-EAS
Frequency	0.0002	-	0.000008	0.000004	0.000054
Homozygous Count	-	-	0	0	0

In Silico Predictions:

Missense Prediction			Splicing Prediction			Nucleotide Conservation Prediction		
**SIFT**	**Mutation Taster**	**CondeI**	**Splice AI**	**dbsc SNV _RF**	**dbscSNV_ ADA**	**PhyloP Vertebrates**	**PhyloP Placental Mammals**	**GERP++**
Deleterious	Disease Causing	Deleterious	Polym orphic	-	-	Conserved	Not Conserved	Conserved

Disease Introduction
Disease Gene: *MYL3*
Disease Name: Familial Hypertrophic Cardiomyopathy type 8 (OMIM:608751)
Inheritance Pattern: Autosomal Dominant or Autosomal Recessive. Heterozygous, homozygous, or compound heterozygous pathogenic variants can cause the disease.
Disease Characteristics: Familial Hypertrophic Cardiomyopathy type 8 is a subtype of Familial Hypertrophic Cardiomyopathy (HCM). HCM is a familial inherited heart disease characterized by asymmetric ventricular hypertrophy, often involving the interventricular septum. Patients are prone to various symptoms triggered by exercise, including dyspnea, syncope, falls, palpitations, and chest pain. The disease shows variable expressivity within and between families, ranging from benign to malignant, with a high risk of heart failure and sudden cardiac death. Phenotypic heterogeneity is significant among individuals. (Reference: OMIM)

Secondary Findings

**Secondary Findings include: Other variants related to the clinical presentation.

Result Interpretation:
No other variants related to the clinical presentation were detected.

Mitochondrial Gene Test Results

**Mitochondrial Test Results include: Reported clearly pathogenic variants related to the clinical phenotype, referencing the human mitochondrial genome database: https://www.mitomap.org
**Mitochondrial genome reference sequence: NC_012920

Result Interpretation:
No other variants related to the clinical phenotype were detected.

Large CNV Test Results

**Chromosomal Test Results include: Chromosomal aneuploidy and triploidy; Pathogenic or likely pathogenic chromosomal copy number variants (CNV) larger ≥ 1Mb.

Result Interpretation:
No pathogenic or likely pathogenic chromosomal CNV variants related to the proband's phenotype were detected.
CNV detection based on high-throughput sequencing is not a routine part of WES analysis and cannot exclude false positives or mosaicism. For more accurate chromosomal CNV results, related chromosomal testing is recommended.

Incidental Findings

**Incidental Findings include: Pathogenic or likely pathogenic variants in the SecondaryFinding_Var database, for which the proband provided informed consent for reporting. Diseases associated with these variants may be unrelated to the current clinical presentation and diagnosis.

Result Interpretation:
No pathogenic or likely pathogenic incidental variants were detected (SecondaryFinding_Var database).

References

1. Emily Olfson, Catherine E Cottrell, Nicholas O Davidson, et al. Identification of medically actionable secondary findings in the 1000 genomes[J]. PloS one, 2015, 9: e0135193.
2. Roddy Walsh, Kate L Thomson, James S Ware, et al. Reassessment of Mendelian gene pathogenicity using 7,855 cardiomyopathy cases and 60,706 reference samples[J]. Genetics in medicine : official journal of the American College of Medical Genetics, 2017 ,2: 192-203.
3. Yubao Zou, Jizheng Wang, Xuan Liu, et al. Multiple gene mutations, not the type of mutation, are the modifier of left ventricle hypertrophy in patients with hypertrophic cardiomyopathy[J]. Molecular biology reports, 2013 ,6: 3969-76.
4. McGurk KA, Zhang X, Theotokis P, et al. The penetrance of rare variants in cardiomyopathy-associated genes: A cross-sectional approach to estimating penetrance for secondary findings[J]. American journal of human genetics, 2023 ,110(9): 1482-1495.

Recommendations

It is recommended that the clinician comprehensively analyzes these test results in conjunction with the patient's clinical symptoms and family verification results.

Testing Methodology and Limitations

Testing Methodology:
This method uses genomic DNA extracted from the proband's blood, saliva, or other tissues as the testing material. First, the DNA is fragmented and a library is prepared. Then, the exonic regions of target genes and adjacent splicing regions are captured and enriched using the Roche KAPA HyperExome panel. Finally, variants are detected using the MGISEQ-2000 or DNBSEQ-T7 sequencing platforms. Quality control metrics for sequencing data are: average sequencing depth of the target region ≥200X, with >98.5% of bases in the target region having a depth >20X.

Data Analysis:

Sequencing reads are aligned to the UCSC hg19 human reference genome using BWA, followed by duplicate removal. SNVs, INDELs, and genotyping are performed using GATK with base quality score recalibration. Exon-level copy number variation (CNV) detection is performed using ExomeDepth.

Variant Filtering and Interpretation:

Gene nomenclature follows the HUGO Gene Nomenclature Committee (HGNC) guidelines; variant nomenclature follows the Human Genome Variation Society (HGVS) guidelines. Variants are annotated and filtered based on the proband's clinical information, population databases, disease databases, and bioinformatic prediction tools (referenced databases and versions are listed in the Appendix). Variant pathogenicity classification follows the guidelines from the American College of Medical Genetics and Genomics (ACMG) and the Association for Molecular Pathology (AMP) (Appendix), incorporating refinements from the ClinGen Sequence Variant Interpretation Working Group and the Association for Clinical Genomic Science (ACGS), among others.

Limitations:

Given the current limitations of medical testing technology and individual variations among subjects, this test cannot guarantee 100% accuracy or a 100% success rate. This method is suitable for detecting point mutations (accuracy >99%) within the captured exonic regions (excluding non-coding regions such as promoters) and the intronic regions adjacent (±20bp) to exons, small insertion/deletion (indel) mutations (<20bp; accuracy >99%), deletion/duplication variants spanning two or more consecutive exons (This method may have a certain false-negative rate due to factors such as genomic region structure, exon size, GC content, the presence of repetitive sequences or regions of high homology, and exonic coverage). Additionally, this method can detect intronic variants documented as pathogenic in the ClinVar and HGMD databases. This technical method can detect point mutations and small indels (<20bp) in the mitochondrial genome but cannot detect changes in mitochondrial DNA copy number. For mitochondrial variants, only the 161 variants included in the BGI internal database with a mutation rate ≥10% are reported; variants below this reporting threshold may be reported at the laboratory's discretion based on the variant's significance. This method can indicate other variant types potentially related to the subject's phenotype, including large genomic copy number variations (CNVs) (e.g., deletions/duplications ≥1Mb), chromosomal aneuploidies, triploidy, loss of heterozygosity (LOH) (≥5Mb), and pathogenic variants involving eight specific dynamic mutation disorders. The accuracy for these results is relatively lower, and they are provided for clinical reference only.

This method cannot detect large genomic CNVs (deletions/duplications <1Mb) and genomic structural variations (e.g., translocations, inversions, LOH <5Mb). This method is not suitable for detecting special types of variants, including but not limited to somatic mutations, deep intronic variants not covered by the exome capture panel, dynamic mutations, gene methylation abnormalities, variants in pseudogene regions, and complex rearrangements. These represent technical limitations inherent to high-throughput sequencing approaches.

Due to the presence of highly repetitive regions, high-GC content regions, complex structural regions, or pseudogenes in some genes, the test may not achieve complete coverage of all exonic regions; however, the overall coverage exceeds 95%. Coverage depth may vary across different genes and exonic regions and may not reach 100% due to capture efficiency (list of low-coverage regions: http://db.bgidx.cn/).

Due to the influence of sample type and quality on probe-capture high-throughput sequencing technology, data imbalance may occur, affecting CNV detection. If data imbalance persists after two rounds of

extraction, library preparation, and sequencing, the analysis will be limited to point mutations and small indels (<20bp).
This analysis is based on the proband's chief complaint and focuses on genes with established pathogenic relationships to monogenic genetic diseases in the OMIM database (2024Q1). Genes associated with polygenic susceptibility or complex diseases are not included in this analysis. If a specific gene panel was selected for analysis, genes outside that panel are not analyzed. Gene names for specified panels must conform to the standardized nomenclature used in the test catalog.
Owing to the current limitations in the understanding of human diseases, the failure to detect a specific gene or pathogenic variant that fully explains the subject's clinical phenotype (i.e., a negative result) cannot exclude the possibility of a genetic disorder. The etiology of some diseases may involve other unknown genes, or variant types that are difficult to detect or confirm with this method.
The DNA used in this test is derived from the subject's blood or other somatic cells. The interpretation deviation caused by chimerism cannot be ruled out.

Test Declaration:
This report is limited to pathogenic, likely pathogenic, or variants of uncertain significance (VUS) that are related to the subject's clinical phenotype and do not conflict with the provided family genetic history. The laboratory cannot guarantee the authenticity, accuracy, or comprehensiveness of the clinical phenotype or suspected diseases provided by the physician or the subject. Inaccurate or incomplete clinical phenotypic and family history information carries the risk of imprecise variant reporting. The heritability of specific variants is based on the described family relationships provided to the laboratory and the possibility of non-maternity or non-paternity is not excluded. Information in this report regarding disease association, correlation explanations, diseases associated with gene variants, and clinical disease characteristics is derived from currently available published research results. Interpretation rules reference relevant ACMG guidelines. The determination of variant pathogenicity is based on existing clinical phenotypes, literature reports, databases, and bioinformatics software predictions and is subject to the limitations of the current state of scientific knowledge; it is for reference only.
This report cannot exclude the presence of other pathogenic alterations in the subject's genome that fall outside the detection scope of this project.
Based on the subject's consent regarding incidental findings (secondary findings) in the informed consent form, this report may or may not include variants listed in the SecondaryFinding_Var (V1.1_2020.3) database.

全外显子组测序检测[redacted]人)

23B0580

医院信息			
送检医院	送检医生	门诊号/住院号	科室
-	-	-	-

样本信息							
到样日期	样本编号	样本类型	姓名	关系	性别	民族	出生日期/年龄
2024-11-09	[redacted]	全血	[redacted]	先证者	女	汉	1992-03-12/32
临床表现和家族史	[redacted]	确诊患者；晕厥，呼吸心跳骤停，CPR 术后，32 岁发病。Brugada 综合征？妊娠期糖尿病；无家族遗传病史					
表型匹[redacted]语	-/-						
临[redacted]/重点分[redacted]基因集	-/-						

检测信息			
检测项目	临床全外显子组检测-单人（B 类）	检测编号	DX1616
检测区域	人类基因组中约 2 万个基因的外显子区+线粒体基因组		
检测策略	针对受检者主诉，对 OMIM 数据库收录的明确致病关系基因进行分析。		
检测方法	芯片捕获高通量测序		

检测结论

主要检测结果为：

在家[redacted]肥厚型心肌病 8 型相关的 *MYL3* 基因上检出与受检者表型部[redacted]关的 1 个意义未明变异。

次要[redacted]：

[redacted]与临床表型相关的变异。

线粒体[redacted]测结果为：

未检出与临床表型相关的线粒体基因变异。

Large CNV 检测结果为：

未检出与受检者表型相关的染色体 CNV 致病 /疑似致病变异。

意外发现检测结果为：

未检出意外发现的致病或疑似致病变异（SecondaryFinding_Var 数据库）。

主要检测结果

序号	基因	染色体位置	转录本编号<br>核苷酸变化<br>(氨基酸变化)	基因亚区	基因型	致病性分类	相关疾病/遗传模式	参考文献
1	[redacted]	chr3:46901000	NM_000258.2:c.446T>C(p.Met149Thr)	EX4/CDS4	杂[redacted]	[redacted]未[redacted]	家族性肥厚型心肌病 8 型(OMIM:608751)/AD,AR	[1-4]

**遗传模式：AD 表示常染色体显性遗传，AR 表示常染色体隐性遗传，XL 表示 X 染色体连锁遗传，YL 表示 Y 染色体连锁遗传，UNK 表示 Unknow，即遗传模式未知。
**主要检[redacted]括：与临床表型相关的致病/疑似致病变异；与临床表型相关，且[redacted]相符的临床意义未明变异。

结果

1、检出[redacted]L3;NM_000258.2:c.446T>C(p.Met149Thr) 变异， 已有该变[redacted]关报道[1-4]。依据 ACMG 指南（附录），该变异被判断为意义未明变异，PM2_Supporting，证据项如下：

PM2_Supporting：PM2 降级情况。

人群频率（各数据库版本见附录）：

数据库	千人基因组	ESP6500	ExAC	GnomAD	GnomAD-EAS
频率值	0.0002	-	0.000008	0.000004	0.000054
纯合个数	-	-	0	0	0

软件预测（各预测软件版本见附录）：

错义预测			剪切位点预测			核酸保守预测		
SI[redacted]	Mutation Taster	Condel	SpliceAI	dbscSNV_RF	dbs[redacted]	PhyloP Vertebrates	PhyloP Placetal Mammals	GERP++
有害	有害	有害	多态	-	-	保守	不保守	保守

疾病介绍

致病基因：*MYL3*

疾病名称：家族性肥厚型心肌病 8 型(OMIM:608751)

遗传模式：常染色体显性遗传或常染色体隐性遗传，杂合，纯合或复合杂合致病变异可导致疾病的发生

疾病特点：家族性肥厚型心肌病 8 型是家族性肥厚型心肌病的一种亚型。家族性肥厚型心肌病是一种以心室不对称肥厚并常累及室间隔的一类家族式遗传性心脏疾病。患者很容易因为运动而引发多种症状，包括呼吸困难、晕厥、摔倒、心悸及胸痛等。本病在家族间和家族内部成员之间可表现出从良性到恶性的差异，具有很高的心衰和心源性猝死的风险。不同个体间表型差异较大。（参考来源：OMIM）

次要检测结果

序号	[redacted]因	染色体位置	转录本编号 核苷酸变化 (氨基酸变化)	基因亚区	基因型	致病性分类	相关疾病/遗传模式	参考文献
-	-	-	-	-	-	-	-	-

**次要检测结果包括：其他与临床表型相关的变异。

结果说明：

未检出其他与临床表型相关的变异。

线粒体基因检测结果

序号	[redacted]	核苷酸变化（氨基酸变化）	突变频率（变异碱基/总碱基 reads 数）	致病性分类	相关疾病 [redacted]	MtD 频率	MITOMAP 频率	参考文献

23B0580

-	[redacted]	-	-	-	-	-	-	-

**线[redacted]括：与临床表型相关的已报道明确致病变异, 参考人类线粒[redacted]库：https://www.mitomap.org
**线粒[redacted]参考序列：NC_012920

结果说明：

未检出其他与临床表型相关的变异。

Large CNV 检测结果

序号	变异名称	片段大小(Kb)	变异来源	致病性分类
-	-	-	-	-

**染色体检测结果包括：染色体非整倍体和三倍体；1Mb 以上的致病或疑似致病的染色体拷贝数变异（CNV）。

结果说明：

未检出与受检者表型相关的染色体 CNV 致病 /疑似致病变异。

[redacted]全外的 CNV 检测不属于全外的常规检测范围，不排[redacted]者嵌合的可能。如需更精准的染色体 CNV [redacted]送检相关染色体检测。

意外发现

序号	基因	染色体位置	转录本编号<br>核苷酸变化<br>(氨基酸变化)	基因亚区	基因型	致病性分类	相关疾病/遗传模式	参考文献
-	-	-	-	-	-	-	-	-

**意外发现检测结果包括：受检者知情同意报告 SecondaryFinding_Var 数据库中的致病或疑似致病变异位点，此类变异关联疾病可能与患者目前临床表现及诊断无关。

结果说明：

未检出意外发现的致病或疑似致病变异（SecondaryFinding_Var 数据库）。

参考文献

[1] E[redacted]Catherine E Cottrell, Nicholas O Davidson, et al. Identi[redacted]edically actionable secondary findings in the 10[redacted]es[J]. PloS one, 2015 ,9: e0135193.
[2] Roddy Walsh, Kate L Thomson, James S Ware, et al. Reassessment of Mendelian gene pathogenicity using 7,855 cardiomyopathy cases and 60,706 reference samples[J]. Genetics in medicine : official journal of the American College of Medical Genetics, 2017 ,2: 192-203.
[3] Yubao Zou, Jizheng Wang, Xuan Liu, et al. Multiple gene mutations, not the type of mutation, are the modifier of left ventricle hypertrophy in patients with hypertrophic cardiomyopathy[J]. Molecular biology reports, 2013 ,6: 3969-76.
[4] McGurk KA, Zhang X, Theotokis P, et al. The penetrance of rare variants in cardiomyopathy-associated genes: A cross-sectional approach to estimating penetrance for secondary findings[J]. American journal of human genetics, 2023 ,110(9): 1482-1495.

建议

建议临床医生参考本检测报告，结合受检者临床症状和家系验证结果进行综合分析。

检测方法和局限

检测[redacted]

本方法以受检者血液、唾液或其他组织来源的基因组 DNA 为检测材料，首先将 DNA 打断并制备文库，然后通过 Roche KA[redacted]yperExome 芯片对目标基因外显子及临近剪切区的 DNA [redacted]捕获和富集，最后使用 MGISEQ-2000 或 DNB[redacted]平台进行变异检测。测序数据质控指标为：目标区[redacted]度≥200×，其中目标区平均深度>20× 的[redacted]比例>98.5%。

数据分析：

测序片段通过 BWA 与 UCSC hg19 人类参考基因组进行比对，去除重复。使用 GATK 进行碱基质量值校正 SNV、INDEL 和基因型检测。使用 ExomeDepth 进行外显子水平的拷贝数变异检测。

变异筛选与解读：

基因命名按照人类基因组组织基因命名委员会（HGNC）命名规范；变异命名按照人类基因组变异学会（HGVS）命名规范。基于受检者临床信息、人群数据库、疾病数据库和生物信息预测工具进行变异注释和筛选（参考数据库及版本见附录）。变异致病性分类依据美国医学遗传学和基因组学学会（ACMG）和美国分子病理学会（AMP）序列变异解释指南（附录），并参考 ClinGen 序列变异解读工作组和英国临床基因组科学学会（ACGS）等对该指南细化解读。

检测局[redacted]

[redacted]检测技术水平的限制和受检者个体差异等原因，[redacted]证 100%的准确性以及 100%的成功率。该方法[redacted]可捕获覆盖外显子区（不包括启动子区等非编码区）及[redacted]邻 20bp 的内含子区中的点突变（检测准确率为 99%以上）、小的缺失插入突变（20bp 以内）（检测准确率为 99%以上），以及连续两个外显子水平的缺失重复变异（受相关基因组区域的结构、外显子大小、GC 含量、是否存在重复区域和高度同源性区域及外显子区覆盖度的影响，本方法可能存在一定的假阴机率）。该方法可以额外检测 ClinVar 及 HGMD 数据库记录致病的内含子区变异。该技术方法可检测线粒体基因组上的点突变和 20bp 以内插入缺失变异，不能检测线粒体基因组拷贝数变化。对于线粒体变异，仅报告华大内部数据库收录的 161 个变异，且突变率≥10%的位点；在报告原则下限的变异，也会根据变异的重要性酌情报告。该方法可提示与受检者表型相关的其它变异类型，包括大片段的基因组拷贝数变异（例如缺失/重复区间≥1Mb）、染色体非整倍体、三倍体、杂合性缺失（LOH，区间≥5Mb）、以及 8 种动态突变致病变异，对于这部分结果，因其准确性相对较低，仅供临床参考。

本方法不能检测大片段的基因组拷贝数变异（缺失/重复区间<1Mb）和基因组结构变异（例如易位、倒位、<5Mb 的 LOH）。本方法不适于检测特殊类型变异（包括但不限于体细胞突变、不在全外芯片覆盖范围内的深度内含子变异、动态突变、[redacted]因甲基化、假基因区域变异、复杂重组等，上述情形均为不[redacted]用于高通量检测的技术局限性范畴）。

由[redacted]因存在高重复区域、高富含 GC 区域、复杂结构区域或[redacted]以致检测不能完全覆盖其所有外显子区，但总[redacted]95%以上。受捕获效率影响，不同基因及外显子[redacted]可能无法达到 100%（低覆盖度区域列表链接[redacted]db.bgidx.cn/）。

由于探针捕获高通量测序技术受检测样本类型，检测样本质量的影响，存在数据不均衡而影响拷贝数变异的情况，实验室在两次提取建库测序依然存在数据不均衡的情况下，则只分析点突变和小的缺失插入突变（20bp 以内）。

本次检测按照受检者主诉，对应 OMIM 数据库（2024Q1）中已明确的单基因遗传病致病基因进行分析，多基因易感疾病相关基因及复杂疾病相关基因不包含在此次分析范围内。若受检者选择分析指定基因集，则非指定基因不在本次分析范围。指定基因集的基因命名，需规范使用检测项目目录中的基因名。

限于目前人类对疾病认识水平的局限性， 如未检出能完全解释受检者临床表型的特定基因及致病变异位点 (即阴性结果)，并不能排除受检者存在某种遗传疾病的可能性，因为某些疾病的发病可能与其它未知基因或本方法难以检测到、或无法确定的基因变异类型有关。

本检测所用 DNA 源自受检者血液或其他体细胞样本，不能排除嵌合现象所致的解读偏差。

检测声明：

本[redacted]与受检者临床表型相关且与受检者家族遗传病史不冲[redacted]疑似致病/意义未明变异。实验室无法对医生[redacted]供的临床表型及临床怀疑疾病的真实性、准确性、全[redacted]证。由于临床表型和家族史提供不准确或不完[redacted]本，存在报告位点提报不精准的风险。特定变异的遗传性[redacted]于向实验室描述的家庭关系，非母（源）性

地址：广东省深圳市盐田区北山工业区 11 栋（邮编：518083）客服电话：400-605-6655 网址：www.bgi.com

或非父（源）性的可能性未被排除。

报告[redacted]均为实验室检测数据，仅用于变异检测之目的，仅供临床[redacted]考，不代表最终诊断结果。本报告中所列变异[redacted]受检者发病，是否致病需结合变异影响、遗传方式[redacted]等多因素综合分析。本报告中与疾病相关性、[redacted]明、基因变异关联疾病及疾病临床特征等信息均来自[redacted]道的研究结果，解读规则参考 ACMG 相关指南（[redacted]录），变异致病性的判定依据现有的临床表型、文献报道和[redacted]库及生物信息学软件判定，受科学发展的阶段性限制，仅供参考。

本报告无法排除在该受检者的基因组中存在本项目检测范围之外的其它致病性改变。

根据受检者签署知情同意书中有关意外发现（secondary findings）的情况，本报告将包含或不包含 SecondaryFinding_Var(V1.1_2020.3)数据库中位点。

**本报告采纳 T/SZGIA 4—2018 团体标准。
**本报告结果只对送检样品负责。本中心对以上检测结果保留最终解释权，如有疑义，请在收到结果后的 7 个工作日内与我们联系。

请到相关遗传咨询门诊或生殖医学门诊就诊！

实验操作人： 报告撰写人：饶斌 审核者： 报告日期：2024-12-04

深圳华大医学检验实验室 报告专用章 4403080118104

23B0580

地址：广东省深圳市盐田区北山工业区 11 栋（邮编：518083）客服电话：400-605-6655 网址：www.bgi.com

附录

1．A[redacted][1-17]证据项说明和致病性计算方法

ACM[redacted]项说明(加*证据项为线粒体检测相关证据项)：

PV[redacted]：当一个疾病的致病机制为功能丧失（LOF）时，检出变异[redacted]功能变异（无义突变、移码突变、经典±1 或 2 的剪接突变、起始密码子变异、单个或多个外显子缺失）。

*PVS1：大片段线粒体 mtDNA 缺失，其中至少一个基因完全缺失。评估蛋白质编码基因中的小缺失，无义和移码变异参照更新版 PVS1。

PVS1_Strong：PVS1 降级情况。

PVS1_Moderate：PVS1 降级情况。

PVS1_Supporting：PVS1 降级情况。

PS1：与先前已确定为致病性的变异有相同的氨基酸改变。

PS2：患者的新发变异，且无家族史（经双亲验证）。

PS2_Very Strong：PS2 升级情况。

PS2_Moderate：PS2 降级情况。

PS2[redacted]orting：PS2 降级情况。

[redacted]体外功能实验已明确会导致基因功能受损的变异。[redacted]

*[redacted]porting：可靠的体外或体内实验表明变异破坏了线粒体[redacted]。

PS4：变异出现在患病群体中的频率显著高于对照群体。

PS4_Moderate：PS4 降级情况。

PS4_Supporting：PS4 降级情况。

PM1：位于热点突变区域，和/或位于已知无良性变异的关键功能域。

PM2：ESP 数据库、千人数据库、EXAC 数据库中正常对照人群中未发现的变异（或隐性遗传病中极低频位点）。

*PM2_Supporting：MtDB 数据库和 MITOMAP 数据库中记录的频率<0.00002 (0.002%, 1/50,000)。

PM3：在隐性遗传病中，在反式位置上检测到致病变异。

PM3_Very Strong：PM3 升级情况。

PM3_Strong：PM3 升级情况。

PM3_Supporting：PM3 降级情况。

PM4：非重复区框内插入/缺失或终止密码子丧失导致的蛋白质长度变化。

PM[redacted]错义突变导致氨基酸变化，此变异之前未曾报道，但是[redacted]位点，导致另外一种氨基酸的变异已经[redacted]性的。

*P[redacted]upporting：PM5 降级情况。

PM6：未经父母样本验证的新发变异。

PM6_Very Strong：PM6 升级情况。

PM6_Strong：PM6 升级情况。

PM6_Supporting：PM6 降级情况。

PP1：突变与疾病在家系中共分离（在家系多个患者中检测到此变异）。

PP1_Strong：PP1 升级情况。

PP1_Moderate：PP1 升级情况。

PP2：对某个基因来说，如果这个基因的错义变异是造成某种疾病的原因，并且这个基因中良性变异所占的比例很小，在这样的基因中所发现的新的错义变异。

PP3：多种统计方法预测出该变异会对基因或基因产物造成有害的影响，包括保守性预测、进化预测、剪接位点影响等。

PP[redacted]携带者的表型或家族史高度符合某种单基因遗传疾病。[redacted]IA*批准的(或同等认证的)实验室中检测在肌肉、[redacted]纤维细胞中 ETC#酶活性降低(<20%)。

B[redacted]SP 数据库、千人数据库、EXAC 数据库中等位基因频率>[redacted]变异（若无特异基因或变异的频率阈值矫

23B0580

正）。

*BA1[redacted]级单倍群定义个体中属于同一顶级单倍群的成员的变体或[redacted]MtDB 和 MITOMAP 的频率等位基因频率> 0.01（[redacted]基因频率大于疾病发病率。

* B[redacted]：在MtDB和MITOMAP的频率等位基因频率在0.005 -0.0099（[redacted].5%-0.99%）之间。

BS2：对于早期完全外显的疾病，在健康成年人中发现该变异（隐性遗传病发现纯合、显性遗传病发现杂合，或者X 连锁半合子）。

* BS2：在健康的成年人中，特别是在健康的母系家庭成员中，变异的异质性高于在受影响个体中的相同组织中的异质性。

*BS2_Supporting：BS2降级情况。

BS3：在体内外实验中确认对蛋白质功能和剪接没有影响的变异。

*BS3_Supporting：在杂交研究或单纤维分析中没有功能的影响。

BS4：在一个家系成员中缺乏共分离。

* BS4：缺乏共分离或者在父系成员中共分离。

BP1：已知一个疾病的致病原因是由于某基因的截短变异，在此基因中所发现的错义变异。

BP2：[redacted]性遗传病中又发现了另一条染色体上同一基因的一个已[redacted]变异，或者是任意遗传模式遗传病中又[redacted]条染色体上同一基因的一个已知致病变异。

*[redacted]个体中发现以前已确认是致病的 mtDNA 的其他变异。

BP3：功能未知重复区域内的缺失/插入，同时没有导致基因编码框[redacted]变。

BP4：多种统计方法预测出该变异会对基因或基因产物无影响，包括保守性预测、进化预测、剪接位点影响等。

BP5：在已经有另一分子致病原因的病例中发现的变异。

*BP5：在与核 DNA 相关的疾病中发现的线粒体 DNA 变异。

BP7：同义变异且预测不影响剪接。

致病性计算方法（各致病性等级对应序号间为“或”的关系）：

致病性等级	序号	判断规则
[redacted]Pathogenic	1	PVS+1 个 PS
	2	PVS+2 个 PM
	3	PVS+1 个 PM +1 个 PP
	4	PVS+≥2[redacted]
	5	≥2 个[redacted]
	6	1 个[redacted]M
	7	1 个 PS[redacted]PM +≥2 个 PP
	8	1 个 PS +1 个 PM +≥4 个 PP
Likely Pathogenic	1	PVS+1 个 PM
	2	1 个 PS +1-2 个 PM
	3	1 个 PS +≥2 个 PP
	4	≥3 个 PM
	5	2 个 PM +≥2 个 PP
	6	1 个 PM +≥4 个 PP
Benign	1	BA1
	2	≥2 个 BS
Likely Benign	1	1 个 BS +1 个 BP
	2	≥2 个 BP
[redacted]ertain Significance	1	以上标准[redacted]符合或致病性有冲突

注：[redacted]

主要[redacted]

[1] Richar[redacted], Aziz N, Bale S, et al. Standards and guidelines for the interpretation of se[redacted]ence variants: a joint consensus recommendation of the

American College of Medical Genetics and Genomics and the Association for Molecular Pathology. Genetics in medicine, 2015, 17(5): 405.
[2] 王秋菊, [redacted]平,等.遗传变异分类标准与指南.中国科学:生命科学,2017(06):76-96.
[3] Bies[redacted]arrison S M. The ACMG/AMP reputable source criteria for the int[redacted]f sequence variants. Genetics in Medicine, 2018, 20(12):[redacted]
[4] Abou[redacted]oun AN, Pesaran T, DiStefano MT, et al. Recommendations for interpreting[redacted]e loss of function PVS1 ACMG/AMP variant criterion. Hum Mutat. 2018, 39(11):1517-1524.
[5] Brnich SE, Abou Tayoun AN, Couch FJ, et al. Clinical Genome Resource Sequence Variant Interpretation Working Group. Recommendations for application of the functional evidence PS3/BS3 criterion using the ACMG/AMP sequence variant interpretation framework. Genome Med. 2019, 12(1):3.
[6] Ghosh R, Harrison SM, Rehm HL; ClinGen Sequence Variant Interpretation Working Group. Updated recommendation for the benign stand-alone ACMG/AMP criterion. Hum Mutat. 2018, 39(11):1525-1530.
[7] Oza AM, DiStefano MT, Hemphill SE, et al. ClinGen Hearing Loss Clinical Domain Working Group. Expert specification of the ACMG/AMP variant interpretation guidelines for genetic hearing loss. Hum Mutat. 2018, 39(11):1593-1613.
[8] Gelb B D, Cavé H, Dillon M W, et al. ClinGen's RASopathy Expert Panel consensus methods for variant interpretation. Genetics in Medicine, 2018, 20(11): 1334.
[9] Mester[redacted]hosh R, Pesaran T, et al. Gene-specific criteria for PTEN variant curation:[redacted]mendations from the ClinGen PTEN Expert Panel. Hum[redacted]9(11):1581-1592.
[10] Ke[redacted]aleshu C, Morales A, et al. Adaptation and validation of the ACMG/[redacted]ant classification framework for MYH7-associated inherited cardiomyopathies: recommendations by ClinGen's Inherited Cardiomyopathy Expert Panel. Genet Med. 2018, 20(3):351-359.
[11] Lee K, Krempely K, Roberts ME, et al. Specifications of the ACMG/AMP variant curation guidelines for the analysis of germline CDH1 sequence variants. Hum Mutat. 2018, 39(11):1553-1568.
[12] Brnich SE, Rivera-Muñoz EA, Berg JS. Quantifying the potential of functional evidence to reclassify variants of uncertain significance in the categorical and Bayesian interpretation frameworks. Hum Mutat. 2018, 39(11):1531-1541.
[13] Zastrow D B, Baudet H, Shen W, et al. Unique aspects of sequence variant interpretation for inborn errors of metabolism (IEM): The ClinGen IEM Working Group and the Phenylalanine Hydroxylase Gene. Human mutation, 2018, 39(11): 1569-1580.
[14] McCormick E M, Lott M T, Dulik M C, et al. Specifications of the ACMG/AMP standards and guidelines for mitochondrial DNA variant interpretation[J]. Human Mutation, 2020.
[15] Brandt T, Sack LM, Arjona D, et al. Adapting ACMG/AMP sequence variant classification guidelines for single-gene copy number variants. Genet Med. 2020, 22(2):336-344.
[16] Won[redacted]Chen T, Wang J, et al. Interpretation of mitochondrial tRNA variants[J].[redacted]cs in Medicine, 2020, 22(5): 917-926.
[17][redacted]en T, Schmitt E S, et al. Clinical and laboratory interpretation of mi[redacted]NA variants[J]. Human Mutation, 2020, 41(10): 1783-17[redacted]

参考数据库及预测软件版本如下：

ClinVar(2024-01-07)， ESP6500(V2)， 千人基因组 (Phase3)， GnomAD(r2.1.1)， ExAC(r1)， BPGD*(V2023Q1)， SecondaryFinding_Var*(V2.0.0_20231030)，dbscSNV(1.1)，SpliceAI(1.3), dbNSFP(2.9.1)，SIFT，MutationTaste，Polyphen2，Phylop，GERP等。 *BPGD (BGI-Phoenix genetic database) 是 BGI 的综合性遗传病数据库。该疾病库以 OMIM 数据库的基因-疾病信息为基础，并整合了 OMIM、Genereview、Orphanet、Genetic Home Reference、Uniprot 等数据库内容，主要涵盖基因、疾病名称、遗传方式、临床特征等信息。 *SecondaryFinding_Var(V2.0.0_20231030)是 BGI 的内部变异数据库，包含 73 个基因上的 2701 个致病和疑似致病变异。

线粒体基因参考数据库及预测软件版本如下：

MtDB: http://www.mtdb.igp.uu.se/(2019-12), MITOMAP: https://www.mitomap.org/(2019-12), GnomAD(v.3.1), MitImpact_db(3.0.6), APOGEE(v.1.0), MitoTIP(3.0.6), HmtVAR: https://www.hmtvar.uniba.it(2019-11). dbNSFP(2.9.1)包含 SIFT， MutationTaster， Polyphen2， Phylop，GERP。线粒体基因位点数据库是 BGI 的内部变异数据库，该数据库包含了来源于 Mitomap，MtDB 和文献的 161 个变异位点。

2. 目标区捕获高通量测序参数

[redacted]	原始数据产出（Mb）	目标区长度（bp）	目标区覆盖度	目标[redacted]深[redacted]	目标区平均深度>10X 位点所占比例	目标区平均深度>20X 位点所占比例
[redacted]	39463.46	42836424	100%	601.52	99.92%	99.82%

3. 受检者 OMIM 数据库中已知致病基因上低频变异信息

见附录

注：

本报告参考 hg19 人类基因组版本。

限于篇幅，本表仅列出检测范围内，OMIM 中已知致病基因上的频率低于或等于 1%的变异（参考数据库包括：千人基因组，ExAC，ESP6500，[redacted]AD，BGI 本地数据库）。

*杂合[redacted]此变异位点为纯合变异，Het 表示此变异位点为杂合变异，[redacted]异位点为性染色体半合子变异。

* “.”[redacted]库没有记录。

4. 检测结果相关附图

1	基因	*MYL3*	转录本	NM_000258.2	染色体位置	chr3:46901000	变异信息	NM_000258.2:c.446T>C(p.Met149Thr)
[redacted] 先证者 [redacted] et	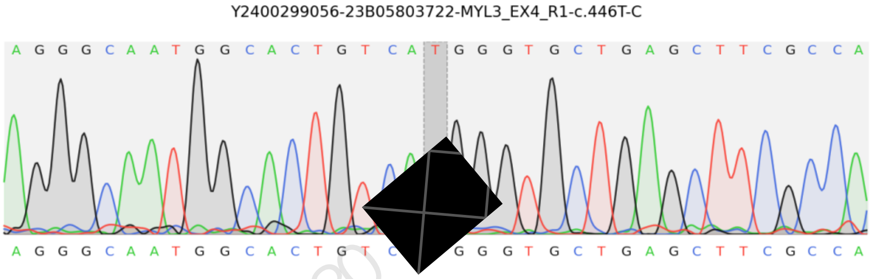							

[redacted]
