# Supplementary figures and images for: Brugada syndrome diagnosed in a young woman occurring postpartum: case report and literature review
Source: Front Cardiovasc Med. 2025 Oct 10;12:1643915. doi: 10.3389/fcvm.2025.1643915 (PMC12549572; doi:10.3389/fcvm.2025.1643915)

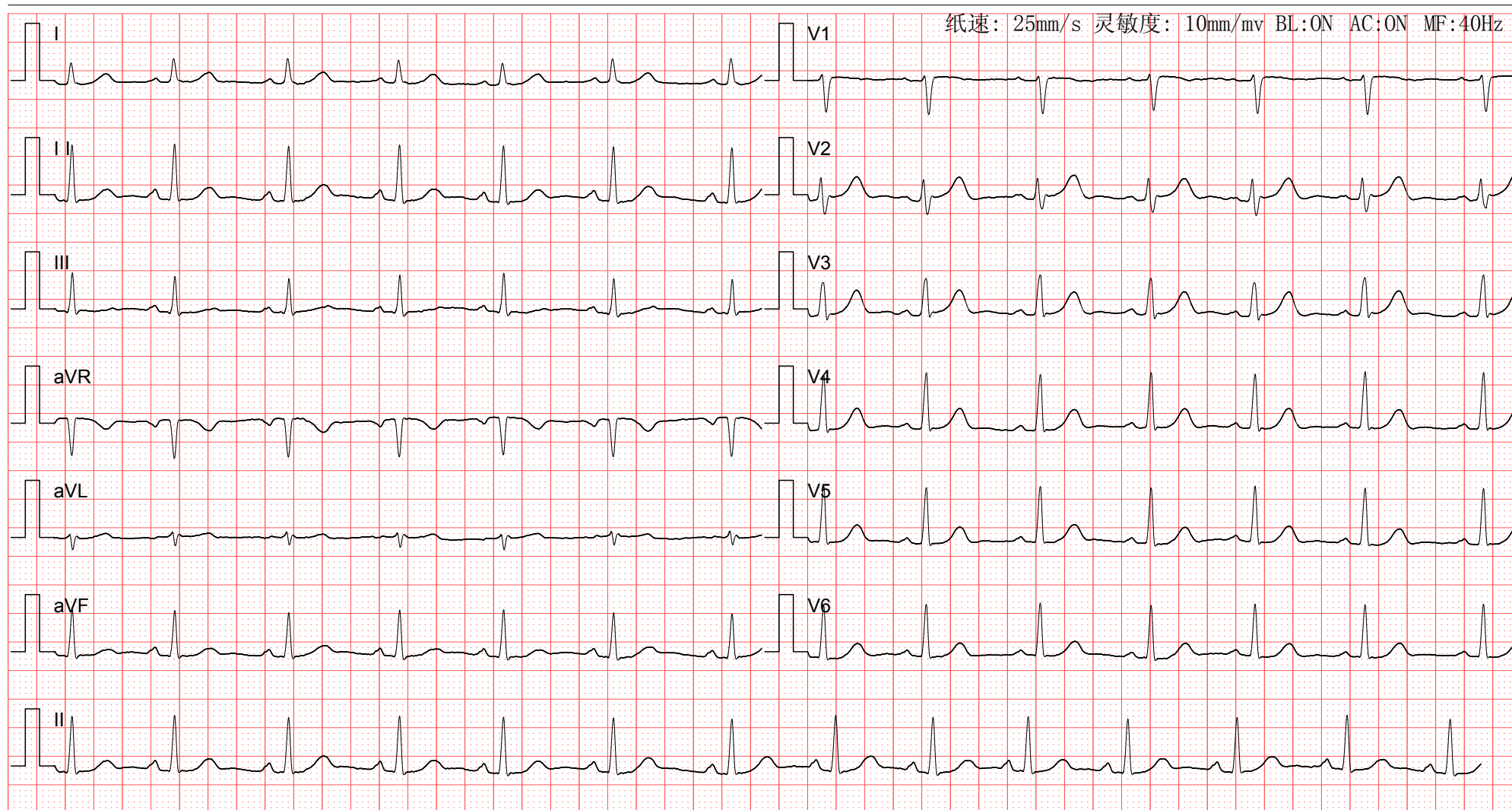

Supplement: Supplementary Figure 1 — The genetic test. [file Image1.pdf]
